# Homograft Aortic Root Replacement for Destructive Prosthetic Valve Endocarditis: Results in the Current Era

**DOI:** 10.3390/jcm13154532

**Published:** 2024-08-02

**Authors:** Marco Pocar, Cristina Barbero, Matteo Marro, Luisa Ferrante, Andrea Costamagna, Luigina Fazio, Michele La Torre, Massimo Boffini, Stefano Salizzoni, Mauro Rinaldi

**Affiliations:** 1 Division of Cardiac Surgery and Cardiothoracic Transplantation, Città della Salute e della Scienza, 10126 Turin, Italy; cristina.barbero@unito.it (C.B.); luisa.ferrante@aeromed.it (L.F.); michele-la.torre@alice.it (M.L.T.); massimo.boffini@unito.it (M.B.); stefano.salizzoni@unito.it (S.S.);; 2 Department of Surgical Sciences, University of Turin, 10126 Turin, Italy; 3 Department of Clinical Sciences and Community Health, University of Milan, 20122 Milan, Italy; 4 Cardiac Intensive Care Unit, Department of Anaesthesia, Intensive Care and Emergency, Città della Salute e della Scienza, 10126 Turin, Italy; 5 Tissue Bank, Città della Salute e della Scienza, 10126 Turin, Italy

**Keywords:** infective endocarditis, aortic root replacement, reoperation, prosthetic valve, homograft, sepsis, organ dysfunction

## Abstract

**Background:** Destructive aortic prosthetic valve endocarditis portends a high morbidity and mortality, and requires complex high-risk surgery. Homograft root replacement is the most radical and biocompatible operation and, thus, the preferred option. **Methods:** A retrospective analysis was conducted on 61 consecutive patients who underwent a cardiac reoperation comprising homograft aortic root replacement since 2010. The probabilities of survival were calculated with the Kaplan–Meier method, whereas multivariable regression served to outline the predictors of adverse events. The endpoints were operative/late death, perioperative low cardiac output and renal failure, and reoperations. **Results:** The operative (cumulative hospital and 30-day) mortality was 13%. The baseline aspartate transaminase (AST) and associated mitral procedures were predictive of operative death (*p* = 0.048, OR [95% CIs] = 1.03 [1–1.06]) and perioperative low cardiac output, respectively (*p* = 0.04, OR [95% CIs] = 21.3 [2.7–168.9] for valve replacement). The latter occurred in 12 (20%) patients, despite a normal ejection fraction. Survival estimates (±SE) at 3 months, 6 months, 1 year, and 3 years after surgery were 86.3 ± 4.7%, 82.0 ± 4.9%, 75.2 ± 5.6, and 70.0 ± 6.3%, respectively. Survival was significantly lower in the case of AST ≥ 40 IU/L (*p* = 0.04) and aortic cross-clamp time ≥ 180 min (*p* = 0.01), but not when excluding operative survivors. Five patients required early (two out of the five, within 3 months) or late (three out of the five) reoperation. **Conclusions**: Homograft aortic root replacement for destructive prosthetic valve endocarditis can currently be performed with a near 90% operative survival and reasonable 3-year mortality and reoperation rate. AST might serve to additionally stratify the operative risk.

## 1. Introduction

Prosthetic infective valve endocarditis (PVE) represents the most severe form of intracardiac infection and is associated with high morbidity and mortality [1]. PVE accounts for 20% of the cases of endocarditis, occurs in up to 6% of patients with a prosthetic valve, and is higher in the presence of bioprostheses [2,3,4]. The etiology and factors favoring infection are different in early versus late PVE [5,6,7,8]. The sensitivity of the Duke criteria is 70–80% for the diagnosis of native valve endocarditis, whereas the 2015 modified criteria and 2023 guidelines of the European Society of Cardiology are more accurate for the diagnosis of PVE [5,6,9].

The aim of surgery for endocarditis is the radical excision of infected tissues and the correction of valvular dysfunction. Despite a trend toward less invasive strategies having been reported to also approach PVE in selected cases, destructive aortic PVE with the erosion of periprosthetic tissues invariably implies a complex, high-risk surgery, often representing a formidable challenge [1,8,10,11,12]. Aortic root replacement may be the sole viable option. The underlying philosophy of homografts is to treat destructive PVE without the further implantation of foreign material, also avoiding rigid prosthetic devices with inherent risks of the dehiscence of fragile infected tissues.

The indications for homografts as the preferred choice to treat more or less extensive endocarditis may vary considerably between centers, coupled with concerns related to durability, particularly in younger patients [13,14,15,16,17,18,19,20,21,22,23,24]. Nevertheless, the essence when opting for a homograft is the improved resistance to infection compared with prosthetic material, particularly within the first six postoperative weeks [18,19,20]. Hence, this approach is considered by most to be the best compromise, if not the gold standard, especially in extensive PVE, and is supported by current guidelines [5]. In this scenario, high-volume single-center experiences have been reported sparingly. Since the pioneering reports during the eighties, mortality has declined but remains non-negligible.

We sought to outline the early and late outcomes, attempting to define the predictors of survival and adverse events.

## 2. Materials and Methods

Retrospective analysis on prospectively collected data was undertaken on 61 consecutive patients who received homograft aortic root implantation to treat destructive PVE at a single institution since 2010. In previous years, homografts had been used sparingly and were less readily available. Data completeness was also inconstantly retrievable in previous years. Exclusion criteria were age < 18 years and cardiogenic shock mandating emergent/salvage operation. Baseline, operative, and perioperative characteristics, depicted in Table 1, were analyzed as potential predictors of adverse events. Hypothesizing that, in spite of elective surgery in relatively stable patients, underlying subtle renal or hepatocellular injury or infection-related systemic inflammatory state might predict a less favorable prognosis, aspartate transaminase (AST), alanine transaminase (ALT), bilirubin, estimated glomerular filtration rate (eGFR), anemia, white blood cell count, platelet count, C-reactive protein, and procalcitonin were included in the analysis. The prognostic role of atrial natriuretic peptides is well-recognized, but data were recorded inconstantly and could not be included.

Although homografts have been indicated more liberally also in patients with native valve endocarditis, we follow a more selective policy and employ homografts almost exclusively in the presence of extensive active infection. Therefore, the indication for homograft implantation was always destructive PVE with involvement of perivalvular structures. All patients presented with prolonged hospitalization and intravenous antibiotic therapy, often with some degree of hepato-renal impairment or heart failure. Nevertheless, no patient in this series underwent salvage surgery in cardiogenic or septic shock, because homografts are unavailable on an emergency basis.

The study complied with the Declaration of Helsinki and was approved by the Institutional Review Board, which waived the need for written informed patient consent.

### 2.1. Operative Technique

**Figure 1:**
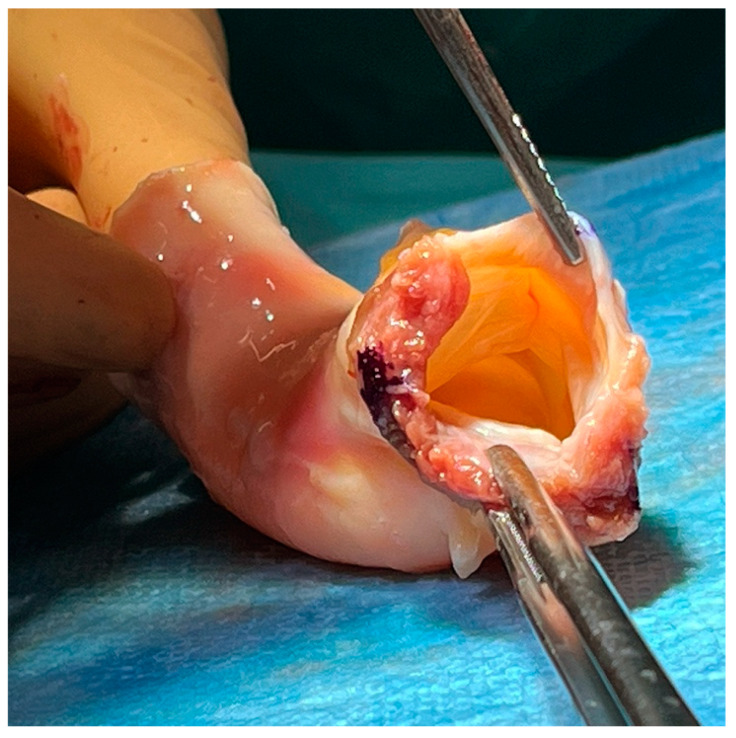
Ventricular aspect of an aortic homograft. Harvesting comprises the entire arch and parts of the left ventricle and mitral valve. Storage is carried out at a tissue bank in liquid nitrogen at −196 °C.

Homografts were always implanted as a full aortic root adopting a button Bentall technique with the aim to achieve radical resection of all infected tissues. The prosthesis is removed and the extension of perivalvular erosion and damage identified. Involved tissues are excised and debrided extensively with subsequent topical disinfection applying iodopovidone and, most often, 0.5% glutaraldehyde. The proximal implant of the homograft on the left ventricular outflow tract is accomplished with 4–0 polypropylene everting mattress sutures buttressed externally with three bovine pericardial strips corresponding to the aortic sinuses. Deep sutures are passed to secure the homograft to healthy tissue. The subaortic curtain, which represents the most common site of erosion and perivalvular abscess in this scenario, is obliterated while retaining the homograft’s subannular tissue and anterior mitral leaflet by placing sutures through the native mitral annulus to reinforce the neo-ventriculoarterial junction (Figure 1). Seldom, a pericardial patch may be necessary for reconstruction, most often in case of septal myocardial erosion or discontinuity. Subsequently, the left and right coronary ostia are sequentially reimplanted with 5–0 running polypropylene sutures and the distal anastomosis completed with 4–0 running suture and external bovine pericardial strip reinforcement. In case of associated mitral valve surgery, repair is preferred when deemed feasible. Incomplete posterior annuloplasty is preferred because it prevents distortion of the subaortic neo-aortomitral continuity. Homografts were stocked prior to surgery in liquid nitrogen at −196 °C and temporarily in dry ice during transport to the operating theatre. The vast majority were provided by our institutional tissue bank.

After defrosting the graft is trimmed as appropriate. The right, left, and non-coronary cusps are visible (lower and upper left, and right position in the image), whereas septal muscle is identifiable on the lower left aspect, opposite to the subaortic curtain and anterior mitral leaflet. During implantation, the latter may be preserved to reinforce the posterior aortic annulus, which represents the most common site of periannular abscess.

### 2.2. Statistical Analysis

Primary endpoints were early and late survival. Secondary endpoints included perioperative complications, namely, low cardiac output, acute kidney injury (AKI), stroke, and cardiac reoperation, mandated or not by recurrent infection. Early operative deaths refer to cumulative hospital and 30-day mortality. Perioperative low cardiac output relates to intravenous epinephrine infusion > 0.05 mcg/kg/min or intra-aortic balloon pump or extracorporeal membrane oxygenation for inotropic and/or mechanical circulatory support, whereas AKI indicates the requirement of new-onset temporary or permanent dialysis, perioperatively. Laboratory test values refer to the closest samples before PVE reoperation. Follow-up is calculated from the day of the operation. Categorical data are presented as frequency (%). Continuous variables are expressed as median [interquartile range, IQR] or mean ± standard deviation (SD), as appropriate. Normality of distribution of continuous variables was assessed with the Shapiro–Wilk test. Univariable analysis of potential predictors was performed by comparison of categorical and continuous variables with the χ^2^, *t,* and U test, as appropriate. Binary logistic regression analysis served to identify predictors of early (operative) adverse events. The probability of late events and differences in probability estimates were calculated with the Kaplan–Meier method and log-rank test. Cox regression analysis was used to identify potential predictors of adverse events over time. The proportional hazard assumption was tested by the result of parallel log(-log(survival) vs. log(time) graphs. All variables listed in Table 1 were tested in the univariable analyses. Multivariable analysis was conducted on covariates demonstrating a trend toward evidence against the null hypothesis at univariable analysis (*p* < 0.15) to identify independent risk factors for adverse outcome events. Significant predictors of early and late death were further adjusted for age, ejection fraction (EF), and eGFR. *p* values < 0.05 were considered significant. SPSS software, version 23, was used for computations.

## 3. Results

### 3.1. Early Mortality and Adverse Events

The operative mortality was 13% (8/61). Two patients died intraoperatively of an uncontrollable hemorrhage at the level of the neo-ventriculoarterial junction related to extreme tissue fragility with the technical impossibility to secure the homograft to the ventricular outflow tract, whereas the cause of death was refractory shock with multiorgan or cardiac failure (two out of six), uncontrolled sepsis (one out of six), or a combination of these conditions in the remaining cases (three out of six). One patient survived the reoperation after temporary mechanical circulatory support with venoarterial extracorporeal membrane oxygenation. A new-onset stroke, most likely of intraoperative cardioembolic origin, occurred in four cases. Not surprisingly, about two-thirds of the patients necessitated a variable degree of higher-dose catecholamine support. A low cardiac output was seen in 12 (20%) cases, in spite of the normal baseline ejection fraction. Almost one-third of the patients developed a complete heart block requiring the implantation of a permanent pacemaker, whereas postoperative AKI with the need for renal replacement therapy was reported in 14 cases (23%).

AST emerged as the sole multivariable predictor of hospital mortality (*p* = 0.048, OR [95% CIs] = 1.03 [1–1.06]), whereas the associated mitral procedures affected a higher probability of perioperative low cardiac output (*p* = 0.007, OR [95% CIs] = 9.4 [1.8–47] for all mitral procedures; *p* = 0.03, OR [95% CIs] = 6.8 [1.2–38] for valve repair; and *p* = 0.004, OR [95% CIs] = 21.3 [2.7–169] for replacement). AST remained predictive of hospital death after the adjustment for age, EF, and eGFR (*p* = 0.004, OR [95% CIs] = 1.04 [1–1.09]). Noteworthily, this resulted when entering AST as a continuous variable. When AST was dichotomized in the analysis, i.e., ≥ or <40 IU/L, the significance was much higher (*p* = 0.01, OR > 10), suggesting an exponential effect of a higher baseline AST on early prognosis. Pre-, intra-, and perioperative variables were analyzed separately because the latter are likely related to the former. However, when entering the perioperative low cardiac output, a well-recognized predictor of hospital death, and AST in a multivariable analysis, both variables maintained a strong trend toward significance in predicting early mortality (*p* = 0.052, OR [95% CIs] = 1.04 [1–1.08] for AST; and *p* = 0.048, OR [95% CIs] = 6.9 [1.06–45.0] for low cardiac output). Conversely, AST was unable to predict early postoperative low cardiac output or AKI, likely in relation to the prevalence of multiorgan failure. Likewise, no other multivariable predictor for AKI was identified.

### 3.2. Late Mortality and Adverse Events

**Figure 2:**
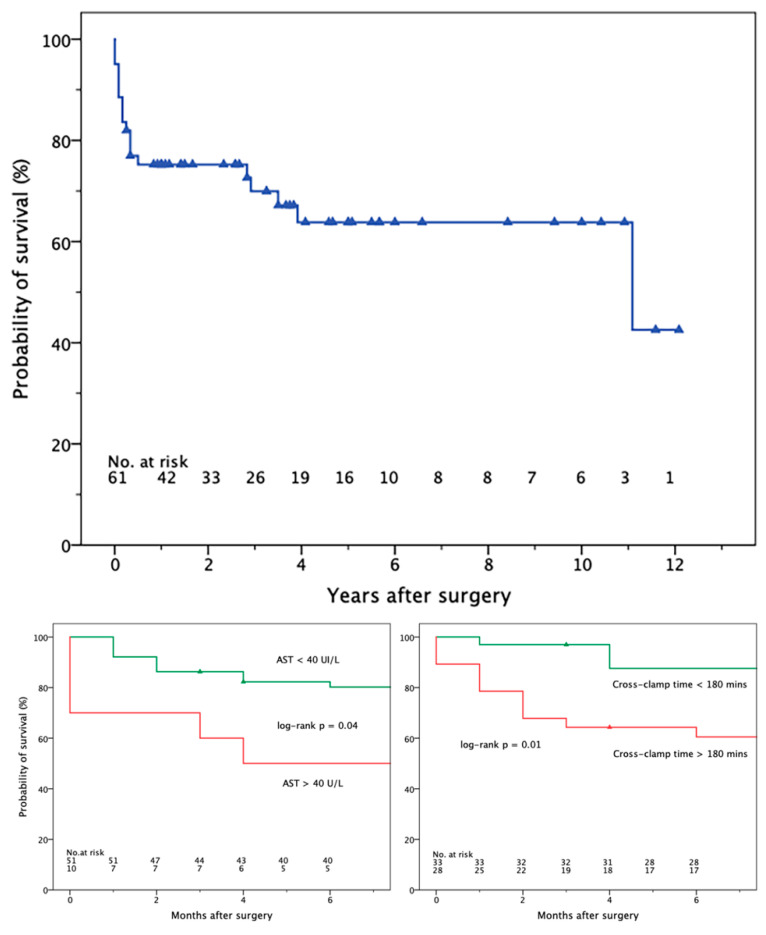
Kaplan–Meier plots depicting the estimated long-term survival (**upper panel**) and 6-month survival according to baseline AST (**lower left**) and myocardial ischemic time (**lower right**). AST, aspartate transaminase.

At a median follow-up of 31 [4–60] months (mean, 39.8 ± 40.7 months), 12 patients died after hospital discharge. In the Kaplan–Meier analysis, the probabilities of survival (±standard error) at 3 and 6 months were 86.3 ± 4.7% and 82.0 ± 4.9%, respectively (Figure 2, upper panel). Thereafter, the probability of late death declined less steeply, with an estimated survival of 75.2 ± 5.6 and 70.0 ± 6.3%, 1 and 3 years after surgery. The survival estimates were significantly lower in the case of AST ≥ 40 IU/L (log-rank *p* = 0.04) and aortic cross-clamp time ≥ 180 min (*p* = 0.01), but not when excluding operative mortality. The corresponding Kaplan–Meier plots, limited to the first 6 postoperative months, are shown in Figure 2, lower panels. No multivariable predictors of late death or adverse events could be outlined when confining the analysis to operative survivors.

In over 80% of the cases, the causes of death at follow-up were major cardiovascular or cerebrovascular events, namely, heart failure, recurrent endocarditis, ischemic/hemorrhagic stroke, or a combination of the aforementioned events.

Five patients underwent a further reoperation, with one operative death. Recurrent endocarditis was the indication in four cases, whereas one patient required a mitral valve replacement in a previous repair at the time of homograft implantation. Reoperation was necessary within the first three months in two patients. Early reoperations were dictated by severe aortic regurgitation due to recurrent endocarditis, and massive mitral regurgitation related to valve repair failure. Further surgeries were performed at 13, 18, and 68 months. One was related to relapsing fungal endocarditis. The sole death occurred after additional early reintervention. No predictors of reoperation or recurrent PVE or combined survival and reoperation could be outlined.

## 4. Discussion

### 4.1. Survival and Perioperative Complications

We observed a 13% hospital mortality, somewhat less than predicted by the EuroSCORE II (mean, 17.9 ± 11.3%; and median, 13 [10–26]). However, a similar discrepancy with the overestimation of operative risk is not uncommon in higher-risk populations. Our results compare equally or favorably in relation to the previously published experience. Other large-volume centers—including, among others, Berlin, Paris, Houston, Harvard, and Brussels—report operative mortalities ranging between 10% and 25% [13,15,16,17,18,19,20,21,22,23,24]. However, the majority of these reports also comprise a homograft root replacement to treat native valve endocarditis in proportions ranging between one-third and half of the patients. A multicenter study from the United States outlined a 12.3% mortality in a near-equal population of 138 patients with native or PVE, in whom the choice of a homograft was mandated by intravenous drug abuse [16]. Analogous to our results, an Italian report from the University of Verona outlined dismal results in patients undergoing homograft root replacement and concomitant multiple valve surgery [13]. Conversely, two tertiary care American university hospitals—Harvard and Mt. Sinai—have questioned the benefits of homografts compared to other prosthetic substitutes [15,23]. The results, however, pertain to retrospective studies and are biased by the patient selection, favoring homografts in the case of extensive root involvement.

Mortality is undoubtedly high when considering (semi-)elective surgery carried out on relatively stable patients, but was related to technical reasons only in the case of the two intraoperative deaths. Thus, the complexity of a challenging surgical anatomy and the intrinsically high procedural risk from a strictly technical standpoint correlated with early death in a minority of the cases and should not be viewed as a contraindication or predictive of a prohibitive procedure. In our experience, most hospital deaths resulted from a low cardiac output, multiorgan failure, and prolonged sepsis, indicating a reduced multiorgan functional reserve. More particularly, multiorgan and cardiac failure primarily occurred after prolonged and complex operations, often including associated mitral or other procedures, and, consequently, implying longer myocardial ischemic times. The development of an acute postoperative septic state, conversely, depicts how a normal or near-normal white blood cell count or procalcitonin levels may not be predictive of the hazards of intraoperative microbial spread. This further renders prosthetic devices less than ideal in this clinical setting, highlighting once more the advantages of implanting a homograft, namely, the resistance to reinfection and tissue compliance with surrounding structures, above all [18]. Compared to prosthesis stiffness, compliance also greatly enhances the technical feasibility of deep subannular implantation in the left ventricular outflow tract by securing the homograft to healthy septal muscle or to the anterior mitral annulus in the case of annular destruction, thus reducing the tear risk. Conversely, the extension of PVE and periannular abscess did not emerge as predictive of an adverse outcome, most probably in relation to a high, near 70% prevalence.

When considering the high surgical risk and complexity, survival, not surprisingly, also continued to steadily decline after discharge, accounting for a 6-month 20% mortality, further stressing the frailty of patients with PVE and prolonged hospitalization. Thereafter, the survival curve tends to progressively flatten. Although the difference between the survival curves reached a significant significance according to the baseline AST ≥ 40 IU/L (see below), this appears to be predominantly related to the effects of the operation within 6–12 months and may likely depend on the sample size. The follow-up of patients in the subgroup with a higher AST is limited and the true impact of this baseline value on a longer-term prognosis warrants further investigation. Speculatively, the predictive role of AST might pertain also to similar patients with prosthetic (or native) valve endocarditis candidated to different cardiac operations.

The perioperative morbidity was also considerable. Despite the normal baseline systolic function, the associated mitral surgery anticipated a higher incidence of perioperative low cardiac output, as previously outlined by others [13]. This most likely reflects the effects of prolonged myocardial ischemia in patients with a reduced multiorgan functional reserve. Not surprisingly, a low cardiac output was observed in all operative non-survivors. Similarly, AKI developed in a near 25% of the patients and in three of the eight operative deaths.

### 4.2. Reoperation

Not surprisingly, 5/61 (8.2%) patients, i.e., almost one of ten hospital survivors (5/53, 9.4%), required subsequent surgery, indicating a non-negligible risk of reoperation. In this context, a recent report from Columbia University regarding the current results in elective non-redo aortic root replacement in 882 patients treated between 2005 and 2019 outlined a 10-year reoperation rate of 5.9% (52 patients) [25]. The median interval to reintervention was 11 months, outlining a higher prevalence of early reoperation despite endocarditis as the primary indication for surgery in only 36 cases (4.1%). Furthermore, a report from the Leipzig group indicates a hospital mortality of 14.3% after mechanical aortic root replacement for endocarditis in primary operations, with no further deaths at one year [26]. The current results related to primary and reoperative aortic root replacement in the United States analyzing 56,447 patients extracted from the Society of Thoracic Surgeons Adult Cardiac Surgery Database depicted an early mortality of 6.2% and 10.8%, respectively, but the data refer to a global cohort and are not confined to destructive PVE [27]. Hence, when considering that our population only comprises complex reoperations in higher-risk patients, the results are comparable or compare favorably to the previously published reports related to first-time or redo surgery, with or without endocarditis as an indication for operation. On the counterpart, further reoperations in our population might have been denied to patients who are too compromised with a prohibitive risk, which may suggest a possible underestimation of the true hazards of the indications for reoperation at late follow-up. The inference of these data, however, was impossible retrospectively. Finally, no reoperation was related to structural homograft degeneration. Consequently, the reoperation rate may be biased and underestimated, and is likely to increase with a more prolonged follow-up.

### 4.3. Biohumoral Outcome Predictors

Although obsolete, AST was reported in 1954 for the diagnosis of myocardial infarction. Unlike ALT, predominant in the liver, AST is equally distributed in the liver, myocardium, skeletal muscle, kidney, brain, and red blood cells, and may be elevated in various organ injuries or systemic injury. Hepatic dysfunction caused by pathogens, toxins, inflammatory mediators, and antibiotics occurs in a near 35% of septic patients and may, ultimately, progress to liver or multiorgan failure, with an increased risk of death [28,29,30]. A higher AST also increases mortality in patients on hemodialysis [31]. Forty UI/L AST or ALT thresholds define liver injury [32]. A cutoff could not be outlined, including in a receiver operating curve analysis, most likely in relation to the sample size. However, in spite of the near-normal baseline AST, the mildly increased values ≥ 40 IU/L were prognostically relevant and were recorded in only 3/53 (5.7%) operative survivors versus 5/8 (62.5%) non-survivors. In other words, the operative mortality was 6.2% (3/48) and 50% (5/10), respectively, according to AST < or ≥40 UI//L (Fisher’s exact test *p* = 0.002)). Right ventricular dysfunction was not prevalent in our population. Thus, liver congestion did not primarily impact the increased AST or ALT. The latter also correlated with operative death, but was not an independent predictor. Speculatively, AST might act as a less specific “sentinel biomarker” of underlying systemic injury, by reflecting a reduced multiorgan functional reserve, possibly not only in PVE. This pathophysiologic conundrum in fact combines a labile hemodynamic state, and toxemia due to infection and prolonged antimicrobial therapy.

Finally, the C-reactive protein and procalcitonin had beyond healthy values, but were not predictive of an adverse outcome. Both were mildly-to-moderately elevated despite non-emergent conditions, an unsurprising finding with multiple pro-inflammatory stimuli, i.e., prolonged systemic infection, the presence of a prosthetic foreign body, hemolysis, and embolization. The lack of a significant correlation can be explained by the fairly constant increase in inflammatory and infective biomarkers in virtually all cases.

### 4.4. Limitations

The retrospective nature of this study and the sample size are intrinsic limitations of the study. A population of 61 patients might underpower the study from a merely statistical standpoint. Nevertheless, the analysis was carried out on a consecutive series at a single center in recent years and focuses on a very specific condition and critical clinical state, with few published reports. Moreover, the actual opportunity to analyze larger populations with similar characteristics in real-world conditions is scarce, whereas different protocols and approaches toward complex endocarditis at different institutions may limit the strengths of multicenter studies. Finally, the reproducibility of results may further be limited by the referral at an experienced, tertiary care center.

## 5. Conclusions

In the current era, reoperation with homograft aortic root replacement for destructive prosthetic valve endocarditis can be performed with a near 90% expected operative survival, and a reasonable 3-year mortality and reoperation rate at experienced tertiary care centers. In addition to reoperation, active endocarditis, and other traditional risk factors for cardiac surgery intrinsically present in patients with PVE, AST might serve as an adjunctive biomarker to stratify operative risk.

## Figures and Tables

**Table 1:** Baseline, operative, and perioperative characteristics vs. operative mortality (n = 61).

	No./Median	(%) [IQR]	Alive	Dead	*p*
			(n = 53)	(n = 8)	
Age (yrs)	68	[54–76]	68	71	0.15
Female	13	(21)	11	2	0.55
Body mass index (kg/m^2^)	25.0	[22.8–26.8]	24.8	25.8	0.84
Hypertension	42	(69)	37	5	0.45
Diabetes mellitus	14	(23)	12	2	0.25
Smoking	34	(56)	31	3	0.23
Drug addiction	8	(13)	8	0	0.30
EuroSCORE II	13	[9–25]	12	25	0.07
Urgent operation	19	(31)	15	4	0.20
Third operation or more	12	(20)	10	2	0.50
Mechanical aortic prosthesis	18	(30)	17	1	0.25
Prior aortic root replacement	9	(15)	7	2	0.34
Prior mitral operation	9	(15)	7	2	0.34
Coronary artery disease	13	(21)	10	3	0.22
Dialysis	4	(7)	4	0	0.56
Ejection fraction	60	[55–61]	60	60	0.45
Aortic regurgitation > 2+	29	(48)	25	4	0.59
Mitral regurgitation > 2+	16	(26)	15	1	0.32
Preoperative inotropes	4	(7)	4	0	0.56
Periannular abscess	42	(69)	35	7	0.21
Subaortic involvement	30	(49)	26	4	0.63
Ventricular septal defect	6	(10)	4	2	0.17
Embolization	19	(31)	16	3	0.48
Stroke	11	(18)	10	1	0.56
Intracerebral hemorrhage	7	(11)	6	1	0.65
Hemoglobin (g/dL)	11.2	[9.7–12]	11.4	10.8	0.23
White blood cell count (×10^9^/L)	9.0	[6.6–11.1]	9.0	9.8	0.81
Platelet count (×10^9^/L)	196	[122–226]	202	168	0.22
eGFR (mL/min)	60	[39–82]	60	44	0.14
AST	22	[15–32]	21	43	0.02
ALT	19	[12–28]	19	30	0.45
Bilirubin (mg/dL)	1.1	[0.9–1.3]	1.0	1.2	0.21
C-reactive protein (mg/L)	14	[1–18]	14	19	0.18
Procalcitonin (mg/mL)	0.5	[0.26–0.80]	0.50	0.52	0.97
MRSA or MRSE	13	(21)	10	3	0.22
Other Staphylococci	11	(18)	9	2	0.37
Streptococci	14	(23)	12	2	0.29
Enterococci	3	(5)	3	0	0.43
Gram-negative	7	(11)	6	1	0.65
Fungus	4	(7)	4	0	0.56
Culture-negative	9	(15)	9	0	0.26
Intravenous antibiotics (days)	16	[12–21]	14	17	0.41
Homograft diameter (mm)	24	[23–25]	24	23	0.95
Mitral valve surgery	27	(44)	22	5	0.23
Repair	20	(33)	18	2	0.47
Replacement	7	(11)	4	3	0.04
Tricuspid valve repair	3	(5)	2	1	0.35
Subaortic patch repair	7	(11)	6	1	0.65
Cardiopulmonary bypass (mins)	230	[195–290]	225	293	0.03
Aortic cross-clamp (mins)	180	[158–204]	171	209	0.002
Dobutamine > 5 mcg/kg/min	41	(67)	38	3	0.07
Epinephrine > 0.05 mcg/kg/min	12	(20)	8	4	0.04
Norepinephrine > 0.15 mcg/kg/min	28	(46)	23	5	0.26
ICU stay (days)	3	[2–7]	3	11	0.02
Mechanical ventilation (days)	1	[1–4]	1	10	0.001
Postoperative inotropes (days)	4	[3–5]	4	8	0.06
Stroke	4	(7)	2	2	0.05
AKI (new-onset dialysis)	14	(23)	11	3	0.14
Permanent pacemaker	18	(30)	18	0	0.10
Hospital stay (days)	15	[18–26]	15	4	0.03
Death	8	(13)	–	–	–

IQR, interquartile range; eGFR, estimated glomerular filtration rate; AST, aspartate transaminase; ALT, alanine transaminase; MRSA, methicillin-resistant *Staphylococcus aureus*; MRSE, methicillin-resistant *Staphylococcus epidermidis*; ICU, intensive care unit; AKI, acute kidney injury.

## Data Availability

Research data are available upon reasonable request.
